# Expression and Function of Kruppel Like-Factors (KLF) in Carcinogenesis

**DOI:** 10.2174/138920209788921010

**Published:** 2009-08

**Authors:** Christophe Bureau, Naima Hanoun, Jérôme Torrisani, Jean-Pierre Vinel, Louis Buscail, Pierre Cordelier

**Affiliations:** 1 Institut National de la Santé et de la Recherche Médicale Unité 858-I2MR, Institut de Médecine Moléculaire de Rangueil, Département Cancers Epithéliaux, Angiogénèse et Signalisation, 31432 Toulouse Cedex 4 France; 2 Service d’Hépato-Gastroentérologie, CHU Toulouse, France

## Abstract

Krüppel-like factor (KLF) family members share a three C2H2 zinc finger DNA binding domain, and are involved in cell proliferation and differentiation control in normal as in pathological situations. Studies over the past several years support a significant role for this family of transcription factors in carcinogenesis. KLFs can both activate and repress genes that participate in cell-cycle regulation. Among them, many up-regulated genes are inhibitors of proliferation, whereas genes that promote cell proliferation are repressed. However, several studies do present KLFs as positive regulator of cell proliferation. KLFs can be deregulated in multiple cancers either by loss of heterozygosity (LOH), somatic mutation or transcriptional silencing by promoter hypermethylation. Accordingly, KLF expression was shown to mediate growth inhibition when ectopically expressed in multiple cancer-derived cell lines through the inhibition of a number of key oncogenic signaling pathways, and to revert the tumorogenic phenotype *in vivo*. Taken together, these observations suggest that KLFs act as tumor suppressor. However, in some occasion, KLFs could act as tumor promoters, depending on “cellular context”. Thus, this review will discuss the roles and the functions of KLF family members in carcinogenesis, with a special focus on cancers from epithelial origin.

## 1. EXPRESSION AND FUNCTION OF KLF IN GASTROINTESTINAL TRACT TUMORS

### A. Expression and Function of KLF in Intestinal and Colon Tumors

KLF4 or gut-enriched KLF (GKLF) is expressed extensively in the gastrointestinal (GI) tract from where it was first identified to maintain enterocyte differentiation and restrain cell proliferation. In mice, KLF4 level peaks by embryonic day 17 [1]. In the newborn, KLF4 level is higher in colon than in small intestine although the levels in both organs rise with increasing age [1]. In human, KLF4 protein is prominent at the epithelium surface in normal colon, and its expression gradually decreases toward the crypt [2, 3]. In RKO cells (human colon cancer-derived cells), KLF4 stimulates intestinal alkaline phosphatase (IAP) gene expression, through a critical region including the IF-III cis element located within the proximal IAP promoter region [4]. In HCT-116 cells (human colon cancer-derived cells), KLF4 increases urokinase-type plasminogen activator receptor (uPAR) [5]. Inversely, level of KLF4 transcript is significantly decreased in intestine during tumor formation in Min mice, a model of intestinal tumorogenesis, suggesting that KLF4 may play a role in gut development and/or tumor burden [1, 6]. KLF4 mRNA and protein levels are significantly and gradually decreased in the dysplasic epithelium of colon, as in adenomatous polyp and colorectal cancer (CRC) [2, 3, 7]. In familial adenomatous polyposis (FAP) patients, KLF4 transcript level is lower in adenomas compared to paired normal-appearing mucosa [6].

The molecular mechanisms involved in KLF4 loss of expression are still unclear. The gene encoding KLF4 is localized on chromosome 9q, previously shown to exhibit loss of heterozigosity (LOH) in CRC [8]. LOH in KLF4 is found in 83% of CRC-derived cell lines, and in 20% of CRC [8]. Using methylation-specific PCR, Dang DT *et al.* showed that KLF4 DNA is not methylated in either normal or tumor tissues from adenomas of Min mice [6]. However, KLF4 expression is stimulated by treating CRC-derived cell lines with 5-Aza-2-deoxycytidine (5-Aza-dC), implicating underlying aberrant epigenetic modifications [3]. Again, KLF4 is methylated in a subset of tumors and cell lines [8]. Other chemicals are used to modulate KLF expression: 5,5’-DibromoDIM up-regulates expression of KLF4 in CRC-derived cell lines, and may represent a novel class of mechanism-based therapeutic drug for CRC [9]. Several point mutations in KLF4 result in a diminished ability to transactivate antiproliferative genes [8]. In addition, KLF4 expression is stimulated following treatment of HT-29 colon cancer cells with IFN-γ  and Stat-1 recruitment [10]. In FAP, KLF4 is induced upon activation of the adenomatous polyposis coli (APC) gene [11-13], probably *via* CDX2 [11]. KLF4 protein is predominantly expressed in cytoplasm but not in nucleus of CRC-derived cells, suggesting that impaired nuclear translocation of KLF4 contributes to cancer formation [14]. Last, KLF4 expression is inversely correlated to beta-catenin/Tcf pathway in HT-29 cells [15].

KLF4 is required for the terminal differentiation of goblet cells in the mouse intestine. Notch signaling pathway suppresses goblet cell formation and is up-regulated in intestinal tumors. Interestingly, KLF4 promoter activity is inhibited by Notch, which controls goblet cell differentiation in mouse GI tract [16], when Notch signaling suppresses KLF4 expression to support intestinal tumors and CRC progression [17].

KLF4 expression is extensively lost in CRC and a downstream target of tumor suppressor genes, suggesting that its re-expression may counteract CRC progression. In cultured cells, expression of KLF4 is associated with growth arrest [18]. Constitutive expression of KLF4 in human CRC-derived cells decreases colony formation, cell proliferation, cell migration, invasion, and *in vivo* tumorogenesis [19]. As a regulator of gene expression, KLF4 acts trough multiple pathways: (i) induction of p21^WAF1/CIP1^ to maintain the integrity of both G_1_/S [20] and G_2_/M [21] cell cycle checkpoints following DNA damage, reduction of cyclin D1 activity by interaction with the Sp1 binding domain on its promoter [22], (ii) repression of beta-catenin expression [12], and (iii) inhibition of ornithine decarboxylase promoter activity through a target, GC-rich, region [23]. Of importance, KLF4 exerts a global inhibitory effect on protein and cholesterol biosynthesis [24]. Interestingly, recent studies suggest that KLF4 functions as an activator or repressor of transcription depending on whether it interacts with co-activators such as p300 and CREB-binding protein or co-repressors such as histone deacetylase HDAC3 [25]. Taken together, these studies clearly demonstrate that KLF4 plays an essential role in the regulation of cell growth in the colon.

Additional KLF family members modulate colon-derived cells proliferation. Despite binding to similar, if not identical, cis-acting DNA sequences, KLF4 and KLF5 often exhibit opposite effects on regulation of gene transcription and cell proliferation [26]. KLF5 promotes proliferation of intestinal epithelia in response to lysophophatidic acid (LPA) [27]. KLF5 protein expression correlates with activating KRAS mutations in intestinal tumors *in vitro* and *in vivo* [28]. Also, KLF5 modulates p53-independent apoptosis through Pim1 survival kinase [29]. Targeting KLF5 results in cell proliferation, transformation and multi-cellular tumor spheroids (MCTS) inhibition [28, 30]. Also, decreasing KLF5 expression using all-trans retinoic acid inhibits proliferation of intestinal epithelial cells [31]. As a consequence, small-molecule compounds are screened on their ability to inhibit the activity of this KLF family member [32]. However, KLF5 becomes anti-proliferative upon TGFβ-mediated acetylation, to maintain epithelial homeostasis [33].

It is less clear how KLF6 expression is regulated in CRC samples. Several studies describe the frequent inactivation by mutation of this putative tumor-suppressor gene [34, 35]. KLF6 mutants lose the ability to induce p21WAF1/CIP1, and fail to inhibit CRC-derived cells growth [34]. LOH of KLF6 locus at chromosome 10p15 contributes to the development of non-polypoid colorectal carcinoma [36], and in the invasion step from an intra-mucosal to an invasive carcinoma [37]. On the other hand, these findings are challenged by the absence of somatic mutations in DNA extracted from frozen tissue samples from larger series of CRC [38]. Interestingly, IGF-1, a well-known proliferation promoter, induces KLF6 expression in a p53-dependent manner [39]. Last, a recent study demonstrates that KLF9 expression is deregulated in CRC, suggesting that this KLF family member may also be involved in the carcinogenesis of human colorectal cancer [40].

### B. Expression and Function of KLF in Gastric, Esophageal and Bladder Cancers

In addition to colon, KLFs exert opposite effects on cell proliferation in numerous examples; while KLF4 and KLF6 inhibit cell growth, KLF5 stimulates proliferation. In gastric cancer, loss of KLF4 expression in primary tumors is associated with poor survival [41, 42]. In parallel, KLF4 mutant mice show increased proliferation and altered differentiation of their gastric epithelia [42]. Interestingly, KLF5 expression is also reduced in gastric carcinomas [43]. Disrupted KLF4 expression contributes to Sp1 over-expression, and to the development and progression of human gastric cancer [44]. Mechanistic studies indicate that promoter hypermethylation and hemizygous deletion contribute to KLF4 down-regulation in cancer [41]. However, epigenetic alterations of KLF4 in gastric oncogenesis is still debated [45]. Enforced expression of KLF4 results in cell proliferation and tumor growth inhibition [41]. In addition, somatic missense mutations are identified in the activation domain of the KLF6 gene in gastric carcinoma [46]. KLF6 LOH is observed in 16 of 37 informative cases [46]. These data suggest that genetic alterations of KLF6 gene play an important role in the development or the progression of sporadic gastric cancers [46]. KLF6 mutants are demonstrated to increase tumorigenicity *in vivo* [47]. However, a recent study demonstrates that the IVS1 -27G/A polymorphism of KLF6 is not associated with an increased risk of gastric cancer in Korean population [48].

Down-regulation of KLF4 expression is also reported in esophageal cancer [49], while over-expression of KLF5 in esophageal epithelia leads to an increase of proliferation in basal cells, *in vivo* [50]. KLF4 represses the transcription of survivin in esophageal squamous cancer cells, which is in favor of an antiproliferative effect [51]. Last, KLF5 transcription factor is described to play an oncogenic role in a human bladder cancer cell line, *via* increased G_1_ to S phase transition, up-regulation of cyclin D1 expression, phosphorylation of MAPK and Akt proteins, and inhibition of p27 and p15 expression [52].

### C. Expression and Function of KLF in Hepatocellular Carcinoma

KLF6 expression and function have been extensively investigated in hepatocellular carcinoma (HCC). KLF6 was first shown to be frequently inactivated either by LOH and/or inactivating somatic mutations, and that its inactivation contributes to pathogenesis of HCC [53-57]. Whereas wild-type KLF6 decreases cellular proliferation of HCC-derived cells, patient-derived KLF6 mutants do not [54, 55]. Also, these mutants are unable to transactivate p21WAF1/CIP1 promoter [55]. DNA methylation may marginally participate to KLF6 down-regulation in HCC [56, 58]. However, deregulation of KLF6 stability following ubiquitination may alter its tumor suppressor function in UV-B irradiated cell lines [59]. Transgenic expression of KLF6 in the liver results in decreased body and liver size, with decreased hepatocyte proliferation, when KLF6 +/- mice display increased liver mass with reduced p21 expression [60].

However, KLF6 status in HCC is still largely debated. No somatic mutations are found in freshly isolated HCC samples [57, 61,  62], and no difference in KLF6 expression are detected between HCC and adjacent normal liver [57]. However, KLF6 expression is inhibited in macronodules, as compared to cirrhosis and HCC [62]. Finally, KLF6 is essential for HCC-derived cells to evade apoptosis [63, 64]. Together with the results describing KLF6 as a tumor suppressor gene, the latter observations create a paradox and indicate that not only loss, but also enforced expression of KLF6 contribute to tumorigenesis. This phenomenon is still not totally understood, and can be attributed to 'cell-type' specificity. Further studies are needed to identify such cellular factors, for KLF6 to switch from a growth-inhibiting tumor suppressor to a growth-promoting oncogene. In fact, KLF6 alternative splicing that yield a dominant negative, proliferation-prone, KLF6-SV1 isoform is described in HCC [65] and in nonalcoholic fatty liver disease [66]. KLF6 alternative splicing in HCC is promoted by oncogenic *KRAS* [65]. Thus, the discrepancy considering KLF6 expression and function in HCC may reside in the identification of the molecular isoform of this transcription factor.

## 2. EXPRESSION AND FUNCTION OF KLF IN OTHER EPITHELIAL CANCERS

### A. Expression and Function of KLF in Prostate Cancer

KLF6 expression is altered in prostate cancer [67]. LOH analysis reveals that KLF6 allele is deleted in 77% of primary prostate tumors [67]. Again, tumor-derived KLF6 mutants fail to transactivate p21^WAF1/CIP1^, and to impede cell proliferation [67, 68], when wild type KLF6 induces apotosis in prostate cancer cells *via* the ATF3 transcription factor [69]. A germline KLF6 single nucleotide polymorphism (IVS1-27G>A), together with increased transcription of three alternatively spliced KLF6 isoforms is reported in prostate cancer. KLF6 protein variants KLF6 SV1 and KLF6 SV2 are mislocalized to the cytoplasm, antagonize wtKLF6 function, leading to a decreased p21 expression and to an increased cell growth. These variants are up-regulated in tumor versus normal prostatic tissue [70]. KLF6 SV1 is demonstrated to be essential for prostate cancer cell growth and spread [71]. However, significant genetic alterations of KLF6 seem restricted to a minority of high-grade prostate cancers [72]. Again, the finding of a broad loss of KLF6 expression in prostate cancer is strongly challenged by independent studies reporting the infrequent germ-line mutation of KLF6, or the presence of KLF6 IVS1-27G>A polymorphism, in prostate cancer [73-77]. Last, this polymorphism predicts the metastatic behavior of the tumors [78]. Taken together, KLF6 SV1 is a novel therapeutic target for prostate cancer. Selenium supplementation is effective in reducing the incidence of prostate cancer. Interestingly, selenium induces KLF4 expression in two cancer cell lines, derived from either androgen-dependent or androgen-independent prostate cancer [79]. Over-expression of KLF4 not only leads to an induction of apoptosis in control cells, but also enhances the DNA-suppressive and pro-apoptotic activities of selenium [79]. Thus, KLF4 may also play a key role in prostate cancer control.

### B. Expression and Function of KLF in Breast and Ovarian Cancers

KLF4 expression is associated with breast cancer progression. Increased KLF4 expression is observed in neoplasic cells compared to adjacent uninvolved epithelium [80]. KLF4 is activated prior to invasion through the basal membrane [80]. Its nuclear localization is associated with an aggressive phenotype in early-stage breast cancer [81]. On the other hand, KLF5 is a potential tumor suppressor gene in breast cancer [82]. KLF5 undergoes haplo-insufficiency during breast carcinogenesis, without evidence of neither point mutation, nor promoter methylation nor homozygous deletion [82]. Re-expression of KLF5 in breast cancer-derived cells inhibits colony formation [82]. Unfortunately, a recent study demonstrates that patients with a higher KLF5 expression have a shorter disease-free survival and overall survival than those observed in patients with a lower KLF5 expression [83]. Thus, KLF5 expression and/or function in breast cancer remain to be elucidated.

Other KLF family members are involved in breast cancer progression. Tissue factor pathway inhibitor-2 (TFPI-2) is a matrix-associated Kunitz inhibitor that inhibits plasmin and trypsin-mediated activation of zymogen matrix metalloproteinases involved in tumor progression, invasion and metastasis. Its expression is lost in highly invasive breast cancer caused by DNA hypermethylation, and as a consequence, a lack in KLF6 transactivation [84]. Interestingly, KLF6 reduction in an ovarian cancer-derived cell line results in a reduction of E-cadherin expression. Conversely, KLF6-SV1 silencing strongly up-regulates E-cadherin, which alters ovarian tumor invasion and metastasis [85, 86]. KLF8 plays a critical role in oncogenic transformation and in epithelial to mesenchymal transition [87]. KLF8 negatively regulates E-cadherin expression which promotes human breast cancer invasion and metastasis [88]. In ovarian cancer cells, KLF8 expression is induced by FAK, by activating the PI3K-Akt signaling pathway [89].

### C. Expression and Function of KLF in Pancreatic Cancer

KLF4 is up-regulated in pancreatic intraepithelial neoplasia, a known precursor lesion of pancreatic cancer [90]. Ectopic expression of KLF4 results in cell cycle arrest of pancreatic carcinoma-derived BxPC-3 cells. In addition, cell growth is inhibited, as tumor growth and metastasis in an orthotopic mouse model. This anti-proliferative effect is due to the interaction of KLF4 with p27(Kip1) promoter [91]. KLF6 is neither lost nor mutated in pancreatic cancer, but accumulates in cytoplasm of the cancer cells caused by an alternative splicing [92]. Interestingly, KLF6 splicing correlates with tumor stage and survival, and may be useful as a prognostic factor.

### D. Expression and Function of KLFs in Lung Carcinoma

Lung KLF (LKLF) is identified as a transactivator of cytosolic phospholipase A2 (cPLA2) in lung epithelial and non-small-cell lung cancer (NSLC) cells [93]. Interestingly, expression of a dominant negative form of LKLF inhibits the induction of cPLA2 promoter activity by *RAS* [93]. Because cPLA2 is critical for increased eicosanoid production associated with lung tumorogenesis, targeting LKLF may be of interest to treat NSLC. In addition, NSLC exhibit frequent allelic loss at chromosome 10p15 where KLF6 is located. Although somatic mutations are not detected in the coding sequence of KLF6, expression of KLF6 mRNA is down-regulated in 85% of primary tumors [94]. Thirty-four percent of informative samples had LOH in the KLF6 gene locus, without evidence of promoter hypermethylation [94]. Enforced expression of KLF6 resulted in NSLC-derived cells apoptosis [94], while PKC activates KLF6 to mediate lung cancer-derived cell lines growth inhibition [95]. In addition, a potential involvement of KLF6 IVS1-27G>A polymorphism is associated with lung cancer risk [96]. Again, an increased expression of KLF6 SV1 is observed in lung adenocarcinoma, and is associated with a decreased survival in patients. In lung cancer cell lines, KLF6 SV1 protects cells from apoptosis, and its targeted reduction induces apoptosis [94, 97, 98].

### E. Expression and Function of KLF in Skin Cancers

In melanoma cell lines, KLF4 expression is down-regulated as a result of homozygous deletion of the CDKN2A locus [99]. A similar study identifies an up-regulation of KLF5 in three melanoma cell lines with the most common and potent V600E mutation in B-RAF gene [100]. Several studies illustrate the function of KLF family members in skin squamous cell carcinoma (SCC). When induced in basal keratinocytes *in vitro*, KLF4 abolishes the distinctive properties of basal and parabasal epithelial cells [101]. Nuclear KLF4 causes a transitory apoptotic response and the skin progresses through phases of hyperplasia and dysplasia [101]. By 6 weeks, lesions highly resemble SCC [101]. KLF4 expression is later demonstrated to be associated with human skin SCC progression, as constitutive nuclear KLF4 staining pattern is detected in moderately and poorly differentiated tumors and metastasis [102, 103]. Interestingly, retinoid receptors including RXRα can be used to prevent KLF4-mediated transformation and tumorogenesis, *in vitro* and *in vivo* [104].

## 3. EXPRESSION AND FUNCTION OF KLFS IN MISCELLANEOUS CANCERS

### A. Expression and Function of KLFs in Central Nervous System Tumors

Dysfunction of KLF family members can contribute to oncogenesis in the brain. Gliomas are frequent tumors of the central nervous system with a poor prognosis. Because deletion of chromosome 10p15 is one of the most common chromosomal alterations in gliomas, KLF6 LOH is detected in 90% of glioblastoma samples [105], without evidences of KLF6 somatic mutations [105-108]. Mutations in KLF6 sequence are absent in astrocytic tumors [109], in meningiomas [106], and in sporadic pituitary tumors [110]. In addition, mutations of KLF6 are infrequent (5.5% to 11.8%) in anaplastic astrocytomas, in low-grade diffuse astrocytomas and in oligodendrogliomas [108]. Still, KLF6 expression is attenuated in human glioblastomas, when compared to primary astrocytes [105, 111, 112]. KLF6 mutants loose their ability to reduce growth rate of glioma cells [111, 112], whereas KLF6 SV1 expression is increased in glioblastomas to sustain cell proliferation [105].

### B. Expression and Function of KLFs in Hematological Malignancies

KLF4, KLF9 and promyelocytic leukemia zinc finger (PLZF) are involved in maintaining cellular quiescence of naive B cells [113]. However, the accelerated response of memory B cells after activation through CD40 and the B cell receptor correlates with reduced expression of these genes, and the subsequent regulatory effects they exert on the cell cycle [113]. In accordance, KLF4 is a putative tumor suppressor in B-cell malignancies [114], whereas KLF5 confers drug resistance to acute lymphoblastic leukemia by promoting survivin expression [115]. Yasunaga *et al.* identified aberrantly hypermethylated DNA sequences, including KLF4 gene, in adult T-cell leukemia (ATL) [116]. Restoring KLF4 expression in ATL-derived cell lines induces cell death by apoptosis [116]. PLZF implication in hematological malignancies is more complex. PLZF is involved in the production of chimeric proteins formed by chromosomal translocations with retinoic acid receptor alpha to produce a dominant negative transcription factor that can decrease the expression of tumor suppressor genes [117]. In acute promyelocytic leukemia, RARα-PLZF overcomes PLZF to contribute to retinoic resistance [118]. In addition, increased expression of PLZF is a predictor of long-term survival in malignant melanoma patients [119]. The silencing of PLZF in melanoma-derived cells unblocks miR-221/-222 expression which in turn leads to enhanced proliferation [120]. Interestingly, CDK2 phosphorylates PLZF, which impairs PLZF transcriptional repression ability and antagonizes its growth inhibitory effects [121].

## CONCLUSION

KLF family member proteins play a critical role in the growth and metastasis of numerous tumor types, at least in part by regulating the expression of cell cycle genes. Globally, KLF4 and KLF6 are considered as tumor suppressor gene, whereas KLF5 promotes cell proliferation. Family members have different transcriptional properties and can modulate each other's activity by a variety of mechanisms. Since cells can express multiple KLFs, KLF transcription factors build likely a transcriptional network to control cell proliferation. Effects of changes in KLF factors are context-dependent and can appear contradictory, considering differences in the expression profile of family members in various cells. Last, KLF variants may antagonize the function of wild type proteins.
